# Hematopoietic Cell Transplantation for Adenosine Deaminase Severe Combined Immunodeficiency—Improved Outcomes in the Modern Era

**DOI:** 10.1007/s10875-022-01238-0

**Published:** 2022-03-15

**Authors:** Elisabetta Ghimenton, Aisling Flinn, Su Han Lum, Timothy R. Leahy, Zohreh Nademi, Stephen Owens, Eleri Williams, Terrence Flood, Sophie Hambleton, Mary Slatter, Andrew R. Gennery

**Affiliations:** 1 Paediatric Haematopoietic Stem Cell Transplant Unit, Great North Children’s Hospital, Clinical Resource Building, Floor 4, Block 2, Queen Victoria Road, Newcastle upon Tyne, NE1 4LP UK; 2 Translational and Clinical Research Institute, Newcastle University, Newcastle upon Tyne, UK; 3 Department of Paediatric Immunology, CHI at Crumlin, Dublin, Ireland

**Keywords:** Adenosine deaminase–deficient severe combined immunodeficiency, Enzyme replacement therapy, Hematopoietic stem cell transplantation, Treosulfan

## Abstract

Current treatment for adenosine deaminase (ADA)-deficient severe combined immunodeficiency (SCID) includes enzyme replacement therapy (ERT), allogeneic hematopoietic stem cell transplant (HSCT), or ex vivo corrected autologous hematopoietic stem cell gene therapy. Historic data show HSCT survival is superior using unconditioned matched sibling and family compared to matched unrelated and haploidentical donors. Recent improvement in HSCT outcomes prompted us to retrospectively examine HSCT survival and long-term graft function in ADA-SCID transplanted at our center. Thirty-three ADA-deficient patients received HSCT between 1989 and 2020, with follow-up data to January 2021. Chemotherapy conditioning regimens were defined as myeloablative (MAC—busulfan/cyclophosphamide), reduced-toxicity myeloablative (RT-MAC—treosulfan-based, since 2007), or no conditioning. Serotherapy used included alemtuzumab (with or without other conditioning agents) or antithymocyte globulin (ATG). ERT was introduced routinely in 2010 until commencement of conditioning. Median age at HSCT was 3.2 (0.8–99.8) months. Twenty-one (63.6%) received stem cells from unrelated or haploidentical donors. Seventeen (51.5%) received chemotherapy conditioning and 16 (48.5%) received alemtuzumab. Median follow-up was 7.5 (0.8–25.0) years. Overall survival (OS) and event-free survival (EFS) at 8 years were 90.9% (95% CI: 79.7–100.0%) and 79% (55–91%), respectively. OS after 2007 (*n* = 21) was 100% vs 75% before 2007 (*n* = 12) (*p* = 0.02). Three (9.1%) died after HSCT: two from multiorgan failure and one from unexplained encephalopathy. There were no deaths after 2007, among those who received ERT and treosulfan-based conditioning pre-HSCT. Ten (30.3%) developed acute GvDH (3 grade II, 2 grade III); no chronic GvHD was observed. In the modern era, conditioned HSCT with MUD has a favorable outcome for ADA-deficient patients.

**Supplementary Information:**

The online version contains supplementary material available at 10.1007/s10875-022-01238-0.

## Introduction

Adenosine deaminase (ADA) is an enzyme in the purine metabolic pathway that converts adenosine and 2′deoxyadenosine into inosine and 2′deoxyinosine, respectively. Absence of ADA results in accumulation of these toxic metabolites, causing a subtype of severe combined immunodeficiency (SCID) characterized by severe lymphocytopenia affecting T- and B-lymphocytes and NK cells and accounts for approximately 10–15% of all SCID cases, although the proportion is higher in some ethnic groups [1–3]. ADA deficiency is also associated with a variety of non-immunological manifestations involving the nervous, hepatic, renal, and skeletal systems, reflecting the ubiquitous expression of this enzyme [4]. Current treatment options include enzyme replacement therapy (ERT), allogeneic hematopoietic stem cell transplant (HSCT), or ex vivo corrected autologous hematopoietic stem cell gene addition therapy (GT). Typically, ERT is used as a bridge to curative treatment using HSCT or GT and is not recommended as long-term therapy because of suboptimal immune reconstitution [5]. Previous data indicated that overall transplant survival is significantly superior using matched sibling (MSD) and family donors (MFD) (86% and 81%, respectively) compared to matched unrelated (MUD) (66%) and haploidentical donors (HID) (43%) [6]. Overall survival was also shown to be superior in unconditioned HSCT compared to myeloablative conditioned HSCT [6]. More recently, improvement in HSCT survival outcomes, including with MUD and haploidentical donors, has been demonstrated [7]. Although less data are available for GT, reports to date are encouraging. Cicalese et al. demonstrated 100% survival in 18 ADA-SCID patients treated with GT with a median follow-up of 6.9 years [8]. Prior to infusion of gene-transduced cells, reduced intensity cytoreductive conditioning with low-dose busulfan is used to make space in the bone marrow niche. Over 100 patients with ADA deficiency have received GT to date. Although all are alive, 10–20% have needed either to re-start ERT or have received subsequent HSCT or GT [9]. Until recently, European guidelines recommended an unconditioned HSCT using a MSD/MFD, or GT if a MSD/MFD is not available, although the recently published guidelines give the option of conditioning for MSD/MFD donors due to the concerns about long-term immunoreconstitution [10]. A conditioned MUD or HID HSCT is now an acceptable alternative, typically reserved for GT failure or when GT is unavailable. Although outcomes of alternative HSCT donors for ADA-SCID have been disappointing, the data are relatively limited and may improve with further advances in allogeneic HSCT technologies. Concerns remain that lack of conditioning may impair long-term immune reconstitution, with lower T-cell receptor excision circle (TREC) levels detected in unconditioned HSCT patients suggesting limited thymopoiesis which may lead to eventual exhaustion of the T-cell receptor repertoire [6]. In light of the significant recent improvements in transplant care for children with inborn errors of immunity, this retrospective observational study aimed to examine the HSCT survival and long-term graft function in children with ADA-SCID transplanted at a single national center.

## Methods

A retrospective chart review was performed of all children with ADA deficiency who underwent first HSCT at the Great North Children’s Hospital, Newcastle upon Tyne, UK, between September 1989 and October 2020. Clinical and laboratory data were obtained from the transplantation database, patients’ medical files, and laboratory records. Follow-up data to January 2021 were included in the analysis. Written informed consent for inclusion in the study was obtained from patients and/or parents or legal guardians of the patients as per institutional practice for HSCT. ADA deficiency was diagnosed by biochemical assays of ADA activity and molecular genetic analysis. Chemotherapy conditioning regimens were defined as myeloablative (MAC), consisting of busulfan/cyclophosphamide; reduced-toxicity myeloablative (RT-MAC), consisting of treosulfan-based regimen (treosulfan/fludarabine or treosulfan/cyclophosphamide); or no conditioning. Treosulfan-based conditioning was introduced in 2007. Serotherapy used included alemtuzumab (with or without other conditioning agents) or antithymocyte globulin (ATG). Enzyme replacement therapy (ERT) in the form of polyethylene glycol-conjugated adenosine deaminase (PEG-ADA) was introduced routinely in 2010 and was administered at the manufacturers recommended dose until commencement of conditioning.

Gene therapy for ADA-SCID was formally incorporated into European Society for Blood and Marrow Transplantation/European Society for Immune Deficiencies Inborn Errors Working Party guidelines in 2017. Subsequently, we counseled each family who did not have a matched family donor about the possibility of a licensed gene therapy product in Milan, Italy, or, when available, gene therapy via a clinical trial at Great Ormond Street hospital, London.

Primary outcomes of interest were overall survival (OS) and event-free survival (EFS). An event was defined as death, graft failure, or second procedure. Secondary outcomes were graft versus host disease (GvHD) and long-term morbidity. Percentage donor chimerism (in whole blood or myeloid/T-cell/B-cell populations) was used as the primary measure of graft function. Cellular immune reconstitution was assessed by total lymphocyte counts, T-cell subsets, and CD19^+^ B-cells. Humoral reconstitution was measured by levels of serum immunoglobulins and by cessation of immunoglobulin replacement therapy (IRT).

### Statistical Analysis

Continuous variables were described using median and range, while categorical variables were reported using counts and percentages. We used the Wilcoxon rank-sum test for comparison of non-parametric data between groups. Log rank test (for comparisons between two groups) and Cox proportional hazards model (for comparisons among more than 2 groups) were used to analyze predictors of OS, EFS, and post-transplant autoimmunity. Variables included for predictor analysis were year of transplant (before 2007 vs during or after 2007), pre-transplant ERT, donor type, and conditioning regimen. All *p*-values were two-sided, with a level of significance at 0.05. Statistical analyses were performed using STATA 14.0 (StataCorp. 2015. Stata Statistical Software: Release 14. College Station, TX, USA: StataCorp LP).

## Results

### Cohort Description and Transplant Details

Thirty-four children were diagnosed with ADA deficiency during the observation period, including ten after 2017. Two of these ten families opted for gene therapy in London—one child was successfully treated and the other received gene therapy, and subsequently had a bone marrow transplant at our center (data reported here). In total, 33 children received HSCT during the observation period (Table 1). Median age at diagnosis was 1.2 (range of birth—95.6) months, and median age at HSCT was 3.2 (range 0.8—99.8) months. Over half of the cohort received stem cells from matched unrelated donors (*n* = 17, 51.5%). Chemotherapy-conditioned stem cell infusions (*n* = 17, 51.5%) included 5 (29.4%) with busulfan-cyclophosphamide (all pre-2007) and 12 (70.6%) with treosulfan-based conditioning (2007 and subsequently). Ten of 12 (83.3%) treosulfan-based conditioning regimens consisted of treosulfan and fludarabine, one (8.3%) treosulfan with cyclophosphamide, and one (8.3%) had additional thiotepa to ensure engraftment, having failed GT. Five of the 12 (41.7%) children with a MFD or MSD had chemotherapy conditioning: four received treosulfan-based conditioning and one received busulfan-cyclophosphamide with additional ATG. The two HID transplants occurred before 2007 and both received busulfan-cyclophosphamide, one with additional alemtuzumab. Alemtuzumab was given as pre-transplant serotherapy to 16 (48.5%) children: 6 (37.5%) with a MFD/MSD (4 of these had treosulfan-based conditioning), 9 (56.3%) with a MUD, and 1 with a HID. Two patients received unconditioned MSD T-cell-depleted BM grafts by in vitro addition of Campath-1 M and one patient received an unconditioned MUD BM with CD34 selection. These three patients were all pre-2007. ERT was given to 17 (51.5%) children transplanted after 2009. These included six (35.3%) with a MFD/MSD and 11 (64.7%) with a MUD. One child underwent MUD HSCT one year after ex vivo lentiviral GT, for marrow failure with evidence of dysplastic change and 6% lymphoblasts on bone marrow aspirate. The child had been blood/platelet and granulocyte colony-stimulating factor-dependent post-GT.

**Table 1:** Cohort and transplant characteristics

	Overall: *n* = 33 (100%)	Pre-2007: *n* = 12 (36.4%)	Since 2007: *n* = 21 (63.6%)
Median age at diagnosis, *m* (range)	1.2 (0–95.6)	2.4 (0–9.6)	1.3 (0.7–2.4)
Median age at HSCT, *m* (range)	3.2 (0.8–99.8)	3.1 (1.1–11.0)	3.8 (0.8–98.3
Median time from diagnosis to HSCT, *m* (range)	1.6 (0.3–23.4)	0.95 (0.4–3.1)	2.1 (0.3–23.4)
Enzyme replacement therapy, *n* (%)	17 (51.5)	0	17 (81.0)
Donor type			
MFD, *n* (%)	12 (36.4)	4 (33.3)	8 (38.1)
MUD, *n* (%)	17 (51.5)	5 (41.7)	12 (57.1)
MMUD, *n* (%)	2 (6.1)	1 (8.3)	1 (4.8)
HID, *n* (%)	2 (6.1)	2 (16.7)	0
Graft type			
BM, *n* (%)	14 (42.4)	7 (58.3)	7 (33.3)
PBSC, *n* (%)	7 (21.2)	0	7 (33.3)
CB, *n* (%)	12 (36.4)	5 (41.7)	7 (33.3)
Stem cell manipulation			
None, *n* (%)	30 (90.9)	9 (75.0)	21 (100.0)
CAMPATH-1 M, *n* (%)	2 (6.1)	2 (16.7)	0
CD34 selection, *n* (%)	1 (3.0)	1 (8.3)	0
Chemotherapy conditioning			
None, *n* (%)	16 (48.5)	7 (58.3)	9 (42.9)
Bu/cy, *n* (%)	5 (15.2)	5 (41.7)	0
Treosulfan-based, *n* (%)	12 (36.4)	0	12 (57.1)
Serotherapy			
None, *n* (%)	16 (48.5)	10 (83.3)	6 (28.6)
Alemtuzumab, *n* (%)	16 (48.5)	1 (8.3)	15 (71.4)
ATG, *n* (%)	1 (3.0)	1 (8.3)	0
GvHD prophylaxis			
None, *n* (%)	6 (18.2)	6 (50.0)	0
CSA only, *n* (%)	5 (15.2)	4 (33.3)	1 (4.8)
CSA/MMF, *n* (%)	19 (57.6)	0	19 (90.5)
CSA/corticosteroid, *n* (%)	2 (6.1)	2 (16.6)	0
CSA/MTX, *n* (%)	1 (3.)	0	1 (4.8)

*HSCT*, human stem cell transplantation; donor type: *HID* haploidentical donor, *MFSD* matched family/sibling donor, *MMUD* mismatched unrelated donor, *MUD* matched unrelated donor; graft type: *BM* bone marrow, *CB* cord blood, *PBSC* peripheral blood stem cells; *ATG*, antithymocyte globulin; conditioning: *Bu* busulfan, *Cy* cyclophosphamide; GvHD (graft versus host disease) prophylaxis: *CSA* ciclosporin, *MTX* methotrexate, *MMF* mycophenolate mofetil

### Outcomes

**Fig. 1:**
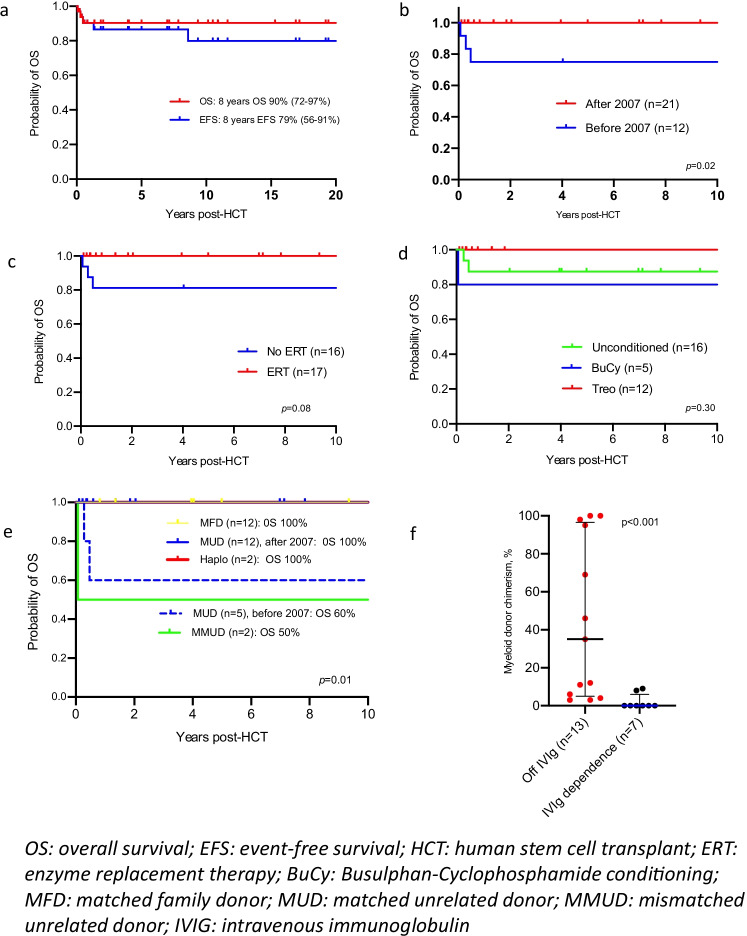
Outcome data after haematopoietic stem cell transplant for adenosine deaminase-deficiency. **a**) Probability in the whole cohort of overall survival (OS) and event free survival (EFS) with an event defined as death, graft failure or second procedure. **b**) Probability of overall survival (OS) in patients transplanted before 2007 or from 2007. **c**) Probability of overall survival (OS) in patients receiving or not receiving pre-transplant enzyme replacement therapy (ERT). d) Probability of overall survival (OS) in patients receiving no pre-transplant conditioning, busulfan-based conditioning or treosulfan-based conditioning. **e**) Probability of overall survival (OS) in patients receiving stem cells from a matched family donor (MFD), matched unrelated donor (MUD), mismatched unrelated donor (MMUD) or T-lymphocyte depleted haploidentical donor. **f**) Relationship between donor myeloid chimerism and immunoglobulin (IVIG) dependence

Median duration of follow-up for surviving patients was 7.5 (range 0.8–25.0) years. The 8-year OS and EFS for the entire cohort were 90.9% (95% CI: 79.7–100.0%) and 79% (55–91%), respectively (Fig. 1a). OS after 2007 (*n* = 21) was 100% compared to 75% before 2007 (*n* = 12) (*p* = 0.02) (Fig. 1b). Three (9.1%) children died after HSCT: two had multiorgan failure and one had unexplained encephalopathy. All three deceased patients received a cord blood (CB) transplant from unrelated donors (two unconditioned; one conditioned with busulfan-cyclophosphamide). There were no deaths in children transplanted after 2007, or among those who received ERT or treosulfan-based conditioning pre-HSCT (Fig. 1c and d). OS was 100% in MFD/MSD, 60% in MUD before 2007 (*n* = 5), 100% in MUD after 2007 (*n* = 12), 50% in MMUD (*n* = 2), and 100% in HID (*n* = 2) (*p* = 0.01) (Fig. 1e). Two additional events were recorded: one patient who initially received unrelated CB had a second HSCT for poor immune function and immune dysregulation; one had stem cell boost infusion for slipping chimerism. Both were alive at the time of data collection. Three (9.1%) children had grade II and two (6.1%) had grade III acute GvHD. No cases of grade IV acute GvHD or chronic GvHD were observed. We did not find significant associations between the development of acute GvHD with donor type, chemotherapy conditioning, year of transplant, or use of ERT.

### Long-Term Outcomes

Of 21 long-term survivors (in follow-up > 2 years post-transplant), median age at last follow-up was 11.1 (range 2.3–25.4) years, with median duration of follow-up 10.9 (range 2.0–25.0) years (Table 2). Median myeloid chimerism in the whole cohort was 6% (range 0–100%), but better in the patients who had received chemotherapy conditioning; median T-cell chimerism was 94% (range 46–100%). Treosulfan-based conditioning was associated with higher median myeloid chimerism at time of last follow-up (82%; range 17–100%) compared to no chemotherapy conditioning (3%; range 0–12%) or Bu-Cy (26%; range 0–98%; *p* < 0.001), whereas graft type (bone marrow vs peripheral blood stem cell vs CB) was not (*p* = 0.4) (Supplementary Fig. S1). Thirteen (65%) discontinued IRT (median myeloid chimerism 13%, range 3–100%; 7 unconditioned, 3 received busulfan-cyclophosphamide, and 3 received treosulfan-based conditioning), while 7 patients remained on IRT (median myeloid chimerism 0%, range 0–9%; 6 unconditioned; one received busulfan-cyclophosphamide) (Fig. 1f).

**Table 2:** Characteristics of long-term survivors (≥ 2 years of follow-up) (*n* = 21)

Median age at last follow-up, yrs (range)	11.1 (2.3–25.4)
Median follow-up, yrs (range)	10.9 (2.0–25.0)
Median myeloid chimerism, % (range) [*n* = 19]	6 (0–100)
Median T cell chimerism, % (range) [*n* = 19]	96 (46–100)
Conditioned HSCT, *n* (%)	7 (33.3)
*-Median follow-up, yrs (range)*	*15.3 (10.9–25.0)*
*-Immunoglobulin dependent, n (%) [Bu-Cy]*	*1 (14.3)*
*-Median latest myeloid chimerism, % (range)*	*46 (0–100)*
Unconditioned HSCT, *n* (%)	14 (66.7)
*-Median follow-up, yrs (range)*	*8.6 (2.0–22.0)*
*-Immunoglobulin dependent, n (%)*	*7 (50)*
*-Median latest myeloid chimerism, % (range)*	*3 (0–12)*
Autoimmunity, *n* (%)	5 (24)

*yrs*, years; *HSCT*, hematopoietic stem cell transplant

**Fig. 2:**
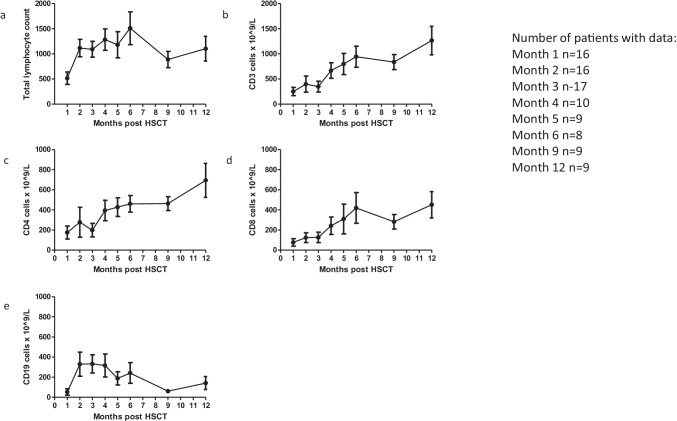
Immune reconstitution post-hematopoietic stem cell transplant for adenosine deaminase deficiency. Total lymphocyte counts (cells/microlitre) over time **a**) Total lymphocyte counts **b**) CD3 count **c**) CD4 count **d**) CD8 count **e**) CD19 count

For children with available data, total lymphocytes and T-cell subset counts increased steadily over time (Fig. 2a–d), approaching levels close to the normal range for age by 12 months post-transplant. In contrast, CD19^+^ B-cell counts decreased over the 9 months post-transplant (Fig. 2e).

The child who had failed GT reconstituted successfully with normal cell counts at the 9-month follow-up.

Five (15.2%) patients developed autoimmunity, two (6.1%) following unconditioned unrelated donor (one MUD and one MMUD) transplants, and three (9.1%) from chemotherapy-conditioned (two busulfan-cyclophosphamide with HID; one treosulfan-based with MUD) transplants: one developed autoimmune hemolytic anemia, hypothyroidism, and type 1 diabetes (HID); one autoimmune hypothyroidism (HID); one juvenile idiopathic arthritis (MMUD); one autoimmune neutropenia (MUD); and one (MUD) had detectable anti-nuclear, anti-double-stranded-DNA, and anti-chromatin antibodies on investigation for mild bilateral ankle pain and facial rash. The latter condition resolved without treatment and has not recurred.

The incidence rate of autoimmunity was 23 cases/1000 person years of follow-up, with a probability of developing autoimmunity by 7.8 years of 23.4% (95% CI 10.0–49.0%), which remained constant to 22 years. Having a mismatched or haploidentical donor was associated with a hazard ratio for the development of autoimmunity of 29.3 (95% CI 2.7–315.6; *p* = 0.005), after adjusting for chemotherapy conditioning and age at transplant.

Two of the five children with post-transplant autoimmunity received alemtuzumab (vs 4/17 with no autoimmunity, *p* = 0.7), and only one had acute GvHD. Median myeloid/T-cell chimerism in children who developed autoimmunity were 6% (range 0–100%)/100% (range 48–100%) vs 17% (range 0–100%)/94% (range 46–100%) for those without autoimmunity (*p* > 0.1 for both comparisons).

One child developed transverse myelitis secondary to echovirus infection in the context of hypogammaglobulinemia post-HSCT. One child developed multiple dermatofibrosarcoma protuberans lesions, which were excised, and the patient remains well at follow-up.

## Discussion

Cure of ADA-SCID requires either allogeneic HSCT or autologous GT, with MSD HSCT followed by GT being the current therapeutic hierarchy, according to European recommendations. However, not all patients have access to either a MSD/MFD or GT, necessitating alternative donor options. Updated European recommendations include unrelated donors as a potential alternative in such cases. We demonstrate that, since 2007, when treosulfan-based chemotherapy conditioning was introduced, outcomes with alternative donor HSCT are comparable to MSD/MFD HSCT, with excellent rates of OS and EFS, low rates of acute GvHD, and no chronic GvHD.

Conditioning in ADA-SCID has been an issue of ongoing debate; patients who have received an unconditioned MSD/MFD HSCT have achieved successful cellular and humoral immune reconstitution [6]. The toxic effects of metabolites that accumulate in ADA deficiency within the bone marrow are thought to cause “auto-conditioning,” providing space to allow donor stem cell engraftment in the absence of chemotherapy. However, the consequences of unconditioned HSCT on the long-term quality of immune reconstitution are not known but may result in limited thymopoiesis leading to eventual exhaustion of the T-cell receptor repertoire [6]. In this cohort, patients who received a chemotherapy-conditioned transplant had superior myeloid chimerism and higher rates of immunoglobulin independence compared to unconditioned patients, also recently demonstrated in the large European experience of SCID transplantation, and another single center report of HSCT for ADA deficiency [11, 12]. Continued follow-up is required to evaluate if this is sustained and to evaluate the impact of higher levels of myeloid chimerism on long-term cellular and humoral immune function. Use of treosulfan-based reduced intensity conditioning in this patient cohort was safe with 100% OS.

Although ERT is not a definitive option in terms of disease correction, use of ERT as a short-term therapy prior to definitive treatment can help to improve endogenous immune function and optimize clinical condition such as in the setting of pulmonary alveolar proteinosis (PAP) [13, 14]. Hassan et al. previously observed no difference in survival outcome between patients who did and did not receive ERT ≥ 3 months prior to HSCT; most patients who received ERT proceeded to have a MUD/MMUD/HID transplant [6]. In contrast, our data support the use of ERT as a bridging therapeutic option, with all patients who received ERT surviving, the majority of whom received a MUD transplant, accepting that this was also for procedures performed in the more recent era, when many other factors relating to pre- and post-transplant care have also improved outcomes. Further evaluation in additional patients is required to determine if it has a survival benefit for all patients or for certain subgroups (for example those with PAP or coexisting infections).

Non-immunological systemic manifestations are well recognized to occur in ADA deficiency, although the underlying pathogenesis is poorly understood, and certain manifestations are only reported in a small number of patients. Neurological, behavioral, and auditory defects do not appear to be corrected by definitive treatment with HSCT or GT. Autoimmunity developed in five patients in this cohort. We identified mismatched and haploidentical donor transplants to be significantly associated with the development of autoimmunity in this cohort. We recently identified the use of alemtuzumab and GvHD as independent risk factors for the development of post-transplant autoimmune cytopenias [15]. However, in this ADA-deficient cohort, neither alemtuzumab nor GvHD was associated with autoimmunity, but numbers of affected patients in this report are too small to draw any definitive conclusions. Further larger studies are needed to determine how factors such as donor type, conditioning, or chimerism might impact outcomes in these areas.

Both OS, particularly since 2007, and EFS in our cohort are comparable to those recently reported for GT for ADA deficiency [16, 17]. Although initial data reported no leukemic change in recipients of gammaretroviral GT [8], a recent report has documented the development of T-cell leukemia in a gammaretroviral GT recipient [18], and persisting prominent clones with vector integration adjacent to proto-oncogenes have been demonstrated in several other patients [19]. Lentiviral GT has demonstrated excellent survival outcomes and safety to date [13], but access to this novel therapeutic modality remains a major limitation. Notably, there is still a place for HSCT in patients who may fail GT, as demonstrated by a case in our cohort.

Limitations of this study include missing long-term cellular immune reconstitution data for patients transplanted pre-2010, and limited follow-up (< 24 m) for a number of children transplanted in the past 2 years. We have not explored the long-term neurodevelopment of these children, a group known to be at risk for poor neuro-behavioral outcomes. As our dataset includes only two HID transplants, more data are needed to determine whether our initial encouraging outcomes using alternative donors will be sustained in the future.

In conclusion, HSCT is a safe curative therapy for children with ADA deficiency, with survival in MUD and HID HSCT comparable to MSD/MFD at this center, and to recent outcome data for GT [8]. HSCT using alternative donors should be considered when no HLA-matched sibling is available, and access to gene therapy is not available. Reduced intensity chemotherapy conditioning and ERT are promising strategies with excellent survival, and conditioned transplants in this cohort resulted in better long-term chimerism and lower rates of IRT dependence. Long-term morbidity in patients with ADA deficiency, particularly autoimmunity and neurodevelopmental outcomes, is an area which requires further study. Further long-term studies are also needed to compare HSCT and GT outcomes.

## Supplementary Information

Below is the link to the electronic supplementary material.

Supplementary file1 (DOCX 23 KB)

Figure S1 Myeloid chimerism by graft type (1a) and by chemotherapy conditioning (1b) (PPTX 54 KB)

## Data Availability

Clinical data files are stored at the Great North Children’s hospital and may be shared according to institutional guidelines.
