# Supplementary material for: Hematopoietic Cell Transplantation for Adenosine Deaminase Severe Combined Immunodeficiency—Improved Outcomes in the Modern Era
Source: J Clin Immunol. 2022 Mar 15;42(4):819–26. doi: 10.1007/s10875-022-01238-0 (PMC9166891; doi:10.1007/s10875-022-01238-0)
Supplement: Supplementary file 1 — Supplementary file1 (DOCX 23 KB) [file 10875_2022_1238_MOESM1_ESM.docx]

**Supplementary Table 1. Transplant characteristics.**

| **ID** | **ERT** | **Donor/graft type** | **Year of transplant** | **Age at transplant (m)** | **Conditioning** | **Dosages** | **Serotherapy** | **Serotherapy dosage** | **GVHD**  **prophylaxis** | **Stem cell manipulation** |
| --- | --- | --- | --- | --- | --- | --- | --- | --- | --- | --- |
| 1 | No | HID BM | 1989 | 4.1 | Bu-Cy | Bu 16; Cy 200mg/kg | Alemtuzumab | 5mg/kg | None | Campath-1M |
| 2 | No | MUD BM | 1996 | 3.1 | Bu-Cy | Bu 16; Cy 200mg/kg | None |  | None | Campath-1M |
| 3 | No | MFSD BM | 1998 | 1.3 | Unconditioned |  | None |  | None | None |
| 4 | No | MFSD BM | 1999 | 1.1 | Unconditioned |  | None |  | None | None |
| 5 | No | MFSD BM | 1999 | 2.9 | Unconditioned |  | None |  | None | None |
| 6 | No | MUD CB | 2000 | 3.2 | Unconditioned |  | None |  | None | None |
| 7 | No | HID BM | 2002 | 4.1 | Bu-Cy | Bu 16; Cy 200mg/kg | None |  | CSA | CD34 selection |
| 8 | No | MUD CB | 2002 | 2.4 | Unconditioned |  | None |  | CSA/corticosteroid | None |
| 9 | No | MUD CB | 2002 | 1.4 | Unconditioned |  | None |  | CSA | None |
| 10 | No | MFSD BM | 2003 | 11.2 | Bu-Cy | Bu 16; Cy 200mg/kg | ATG | 12.5mg/kg | CSA | None |
| 11 | No | MMUD CB | 2004 | 3.7 | Bu-Cy | Bu 16; Cy 200mg/kg | None |  | CSA | None |
| 12 | No | MUD CB | 2006 | 3.4 | Unconditioned |  | None |  | CSA/corticosteroid | None |
| 13 | No | MFSD BM | 2007 | 2.5 | Flu-Treo | Flu 150mg/m2; Treo 36g/m2 | Alemtuzumab | 0.3mg/kg | CSA/MMF | None |
| 14 | No | MUD BM | 2008 | 4.9 | Treo-Cy | Treo 42g/m2; Cy 200mg/kg | Alemtuzumab | 0.3mg/kg | CSA/MTX | None |
| 15 | No | MFSD BM | 2009 | 2.7 | Flu-Treo | Flu 150mg/m2; Treo 36g/m2 | Alemtuzumab | 0.3mg/kg | CSA/MMF | None |
| 16 | Yes | MFSD BM | 2010 | 5.4 | Unconditioned |  | None |  | CSA | None |
| 17 | No | MMUD CB | 2010 | 1.1 | Unconditioned |  | None |  | CSA/MMF | None |
| 18 | Yes | MFSD BM | 2011 | 1.9 | Unconditioned |  | None |  | CSA/MMF | None |
| 19 | Yes | MUD CB | 2011 | 2.9 | Unconditioned |  | None |  | CSA/MMF | None |
| 20 | Yes | MUD CB | 2013 | 3.3 | Unconditioned |  | None |  | CSA/MMF | None |
| 21 | Yes | MUD CB | 2013 | 0.8 | Unconditioned |  | None |  | CSA/MMF | None |
| 22 | Yes | MFSD PBSC | 2015 | 2.9 | Unconditioned |  | Alemtuzumab | 0.3mg/kg | CSA/MMF | None |
| 23 | Yes | MFSD PBSC | 2016 | 1.9 | Unconditioned |  | Alemtuzumab | 0.3mg/kg | CSA/MMF | None |
| 34 | Yes | MUD PBSC | 2018 | 99.8 | Flu-Treo | Flu 150mg/m2; Treo 42g/m2 | Alemtuzumab | 1.0mg/kg | CSA/MMF | None |
| 24 | Yes | MUD PBSC | 2018 | 3.5 | Unconditioned |  | Alemtuzumab | 1.0mg/kg | CSA/MMF | None |
| 26 | Yes | MUD CB | 2019 | 4.7 | Flu-Treo | Flu 150mg/m2; Treo 36g/m2 | Alemtuzumab | 0.6mg/kg | CSA/MMF | None |
| 27 | Yes | MFSD BM | 2019 | 5.7 | Flu-Treo | Flu 150mg/m2; Treo 36g/m2 | Alemtuzumab | 0.6mg/kg | CSA/MMF | None |
| 28 | Yes | MUD CB | 2019 | 5.6 | Flu-Treo | Flu 150mg/m2; Treo 30g/m2 | Alemtuzumab | 0.6mg/kg | CSA/MMF | None |
| 29 | Yes | MFSD BM | 2019 | 3.9 | Flu-Treo | Flu 150mg/m2; Treo 30g/m2 | Alemtuzumab | 0.6mg/kg | CSA/MMF | None |
| 30 | Yes | MUD PBSC | 2020 | 12.8 | Flu-Treo | Flu 150mg/m2; Treo 30g/m2 | Alemtuzumab | 1.0mg/kg | CSA/MMF | None |
| 31 | Yes | MUD CB | 2020 | 4.0 | Flu-Treo | Flu 150mg/m2; Treo 30g/m2 | Alemtuzumab | 0.6mg/kg | CSA/MMF | None |
| 32 | Yes | MUD PBSC | 2020 | 43.3 | Flu-Treo-Thio | Flu 150mg/m2; Treo 36g/m2; Thio 10mg/kg | Alemtuzumab | 1.0mg/kg | CSA/MMF | None |
| 33 | Yes | MUD PBSC | 2020 | 4.5 | Flu-Treo | Flu 150mg/m2; Treo 30g/m2 | Alemtuzumab | 1.0mg/kg | CSA/MMF | None |

*ID: patient number; ERT: enzyme replacement therapy pre-transplant; Donor type: HID – haplo-identical donor, MFSD – matched family/sibling donor, MMUD – mismatched unrelated donor, MUD – matched unrelated donor; Graft type: BM – bone marrow, CB – cord blood, PBSC – peripheral blood stem cells; Conditioning: Bu – Busulfan, Cy – Cyclophosphamide, Flu – Fludarabine, Treo – Treosulfan, Thio – Thiotepa; ATG: antithymocyte globulin; GVHD prophylaxis: CSA – ciclosporin, MTX – methotrexate, MMF – mycophenolate mofetil.*
