# Supplementary figures and images for: Hematopoietic Cell Transplantation for Adenosine Deaminase Severe Combined Immunodeficiency—Improved Outcomes in the Modern Era
Source: J Clin Immunol. 2022 Mar 15;42(4):819–26. doi: 10.1007/s10875-022-01238-0 (PMC9166891; doi:10.1007/s10875-022-01238-0)

## Slide 1
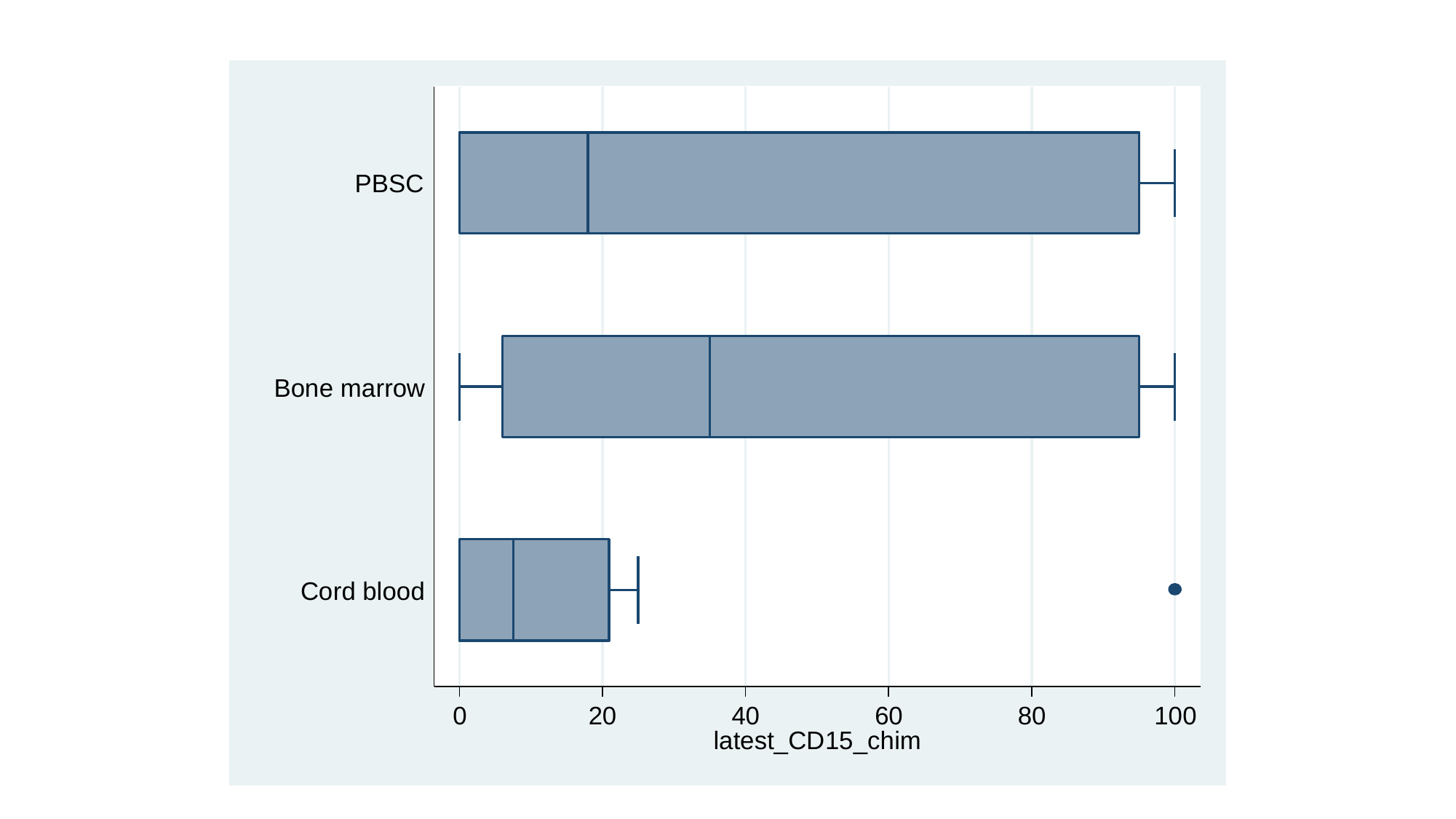

## Slide 2
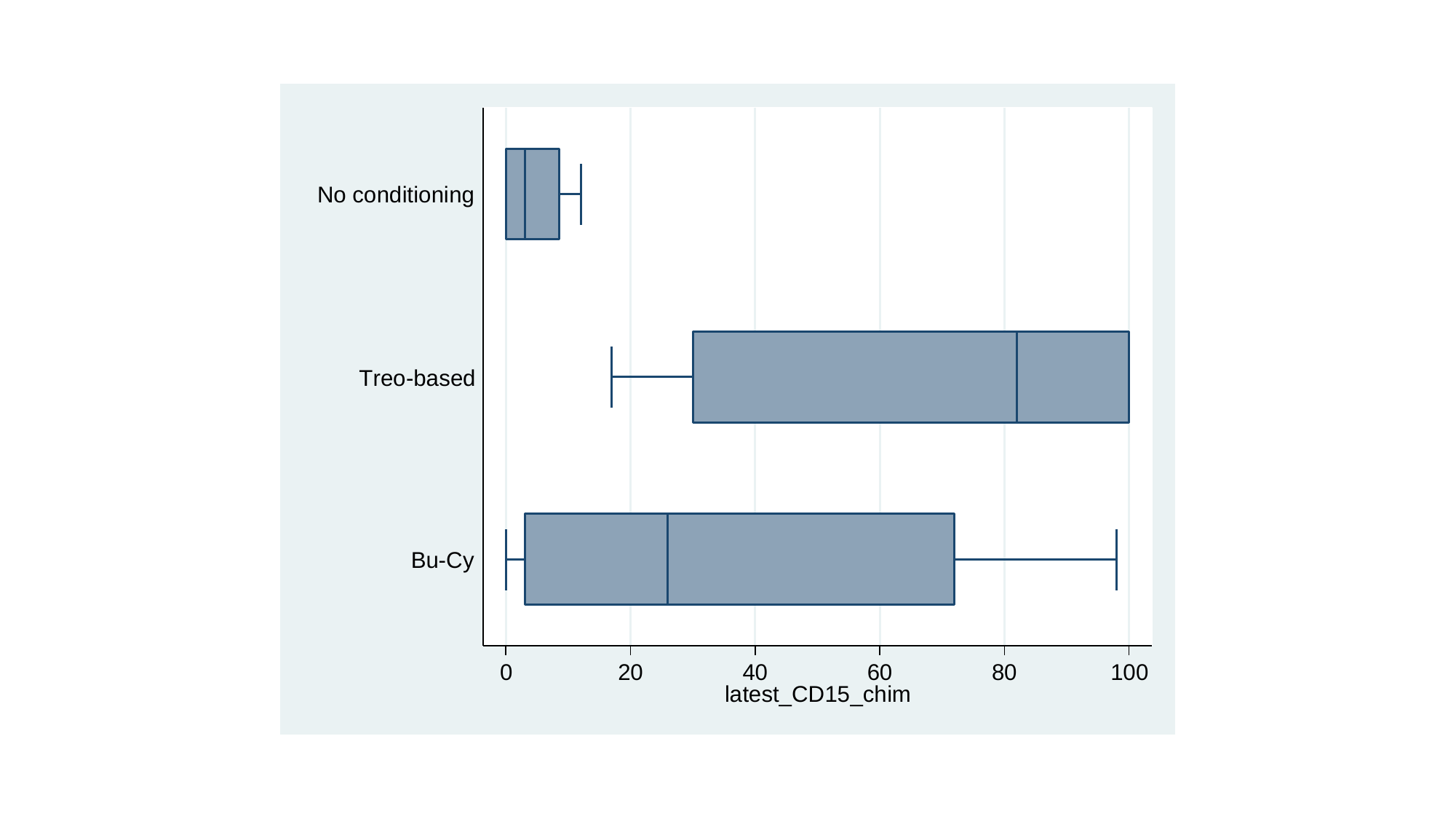

Supplement: Supplementary file 2 — Myeloid chimerism by graft type (1a) and by chemotherapy conditioning (1b) (PPTX 54 KB) [file 10875_2022_1238_MOESM2_ESM.pptx]
